# The Factors Affecting Volunteers’ Willingness to Participate in Disaster Preparedness

**DOI:** 10.3390/ijerph18084141

**Published:** 2021-04-14

**Authors:** Yingnan Ma, Wei Zhu, Huan Zhang, Pengxia Zhao, Yafei Wang, Qiujie Zhang

**Affiliations:** 1 Beijing Research Center of Urban Systems Engineering, Beijing 100035, China; yingnanma@126.com (Y.M.); zhuweianquan@126.com (W.Z.); zhaopengxia2021@163.com (P.Z.); yafeiw@126.com (Y.W.); 2 School of Social Development and Public Policy, Beijing Normal University, Beijing 100875, China; zhanghuan@bnu.edu.cn; 3 Department of Research Project, Beijing Vocational College of Labor and Social Security, Beijing 100029, China

**Keywords:** volunteering, disaster preparedness, accidental life insurance, training, organizational identification

## Abstract

Disaster preparedness is crucial for providing an effective response to, and reducing the possible impacts of, disasters. Although volunteers’ participation plays an important role in disaster preparedness, their actual participation in disaster preparedness activities is still low. To find ways to encourage more volunteers to participate, this study analyzed the social background and organizational and attitudinal factors affecting the volunteers’ willingness to participate. Questionnaires were distributed to 990 registered disaster volunteers across Beijing and the data were analyzed using linear regression models. Results revealed a weak willingness to participate in disaster preparedness. Only 28.08% of the respondents indicated that they were “very ready” to participate in voluntary disaster preparedness, and 14.65% showed “a little bit” of interest. The following was concluded: (1) Disaster volunteers’ social background variables were related to their willingness to participate in disaster preparedness. Compared to male volunteers, female volunteers were more willing to participate. Chinese Communist Party members were more willing to participate than non-members. (2) Providing accidental life insurance for the volunteers had a positive effect on their willingness to participate in disaster preparedness. Provision of more training had a negative effect on the volunteers’ willingness to participate, indicating a low quality of training. (3) Organizational identification was positively related to the volunteers’ willingness to participate. According to these results, we suggest that volunteer organizations should improve their standards and procedures for disaster volunteer recruitment and selection, and gain a deeper understanding of the needs of the disaster volunteers in order to better motivate them to participate.

## 1. Background

Disaster preparedness is a core part of disaster risk management and is crucial for providing an effective response and reducing possible impacts [1]. It is a key indicator of a community’s emergency preparedness system vulnerability (EPSV) [2]. Volunteers’ participation in disaster preparedness is an effective component of a community’s disaster mitigation resources. In the Wenchuan Earthquake in 2008, a variety of people survived because of well-organized disaster relief provided by volunteers. A typical example is provided by the Sang Zao Middle School, where voluntary and regular disaster preparedness activities successfully helped over 2200 faculty and students escape from the school buildings within just two minutes. As a result, not a single person was injured or killed. 

The Wenchuan Earthquake marks the start of the Disaster Volunteering Era in China. Since then, disaster preparedness volunteering has been booming, as indicated by the rapidly increasing number of registered disaster volunteers and organizations across the nation. However, the volunteers’ actual participation in disaster preparedness activities is not proportionately increasing. A survey from China revealed that as few as one-fourth of the respondents, who were primary care health staff volunteers, participated in emergency response in the past [3]. The same phenomenon was found in other countries as well [4,5]. Why are volunteers reluctant to participate in disaster preparedness? Previous studies have explored the determinants of volunteers’ participation in the context of a disaster attack [6], but they have largely ignored disaster preparedness. The current study contributes to the research literature by analyzing the factors affecting volunteers’ willingness to participate in disaster preparedness activities. Moreover, the results will help volunteer organization managers improve the standards and procedures of disaster volunteer recruitment and selection, and help them gain a deeper understanding of the needs of the disaster volunteers to better motivate them to participate in disaster preparedness. 

In this paper, we explore the current status of volunteers’ willingness to participate in disaster preparedness, and then examine the determinants of their willingness to participate. We adopt as our data set a territory-wide survey from Beijing in 2020. Beijing is under constant threat of emerging and re-emerging natural disasters. Located in the North China seismic zone, Beijing is vulnerable to earthquakes. Meanwhile, the rural area of this city, surrounded by the Yan Mountains, faces constant risks of landslides and floods in summer. Thus, voluntary participation in disaster preparedness is very important. There are 140,045 registered disaster volunteers in Beijing, and 71,226 of them belong to 537 volunteer organizations. The organized volunteers are required to provide disaster prevention and mitigation training to local residents, collect and report risk information, and organize regular drills and other disaster preparedness activities for local communities. 

This paper is organized as follows. First, the factors affecting volunteers’ participation are reviewed and summarized. Second, the data collection procedures, the measurements of variables, and the data analysis methods are presented. Third, the results of regression models are reported. Finally, policy implementation and potential theoretical contributions are discussed.

## 2. Theoretical Background and Hypothesis Development

A range of explanations have been offered to account for volunteers’ participation in disaster response. These can be grouped into three main categories: social background variables, organizational variables, and attitudinal variables. 

### 2.1. Social Background Variables

The relation between social background and volunteers’ participation has been discussed relatively thoroughly. The Dominant Status Model of Smith (1994) proposed that people with a higher socioeconomic status are more likely to volunteer [7]. This model has received wide attention [8,9,10,11,12]. Borrowing from this model, Wilson and Musick (1997) established the Resource Model and conceptualized volunteer work as requiring human capital, social capital, and cultural capital [8]. Based on this model, people who have surplus financing and feel secure are more likely to volunteer [13]. Many studies have validated these models, indicating that people with higher education are more likely to volunteer [14,15]. 

Disaster social science has also borrowed from Liberal Feminist Theory [16]. Disaster organizations often hold the stereotypical notions that women’s roles in disaster relief efforts are limited by femininity [16]; however, application studies have revealed complicated results. Some studies have suggested that volunteer participation is greater for males [15,17,18]. Some studies have found that participation can be predicted by a combination of gender and marriage. Andreoni and Payne (2003) found that married males are more sensitive to the price of donating than are married females [19]. Other studies show no significant relationship between gender and volunteering [20]. Moreover, studies on the role of gender in volunteers’ participation under disaster scenarios have been quite limited [21]. One survey showed that female healthcare workers are less likely to deploy in the event of a disaster [22]. 

Political status should also be taken into account. People who are Chinese Communist Party (CCP) members are usually expected to be more devoted to community development; indeed, it has been observed that party members are more willing to volunteer [23]. 

Based on the analysis above, we propose the following hypotheses:

**Hypothesis** **1.** *Male volunteers are more willing to participate in disaster preparedness.*

**Hypothesis** **2.** *Higher education level has a positive effect on the volunteers’ willingness to participate in disaster preparedness.*

**Hypothesis** **3.** *Being a Chinese Communist Party member has a positive effect on the volunteers’ willingness to participate in disaster preparedness.*

### 2.2. Organizational Variables

Volunteering occurs in an organizational context, and thus organizational variables are highly associated with willingness to volunteer [24]. In general, volunteer organizations play a vital role in “pushing” and “pulling” volunteers to participate. On the one hand, a volunteer organization can “push” its members to volunteer by addressing their concerns, such as inadequate capacity and potential physical or mental risks. The Job Demands–Resources (JD-R) model proposes a variety of work-related factors that determine an employee’s well-being [25]. Those factors can be categorized as job demands and job resources. Job demands refers to the characteristics of the job that require the employees’ physical and/or psychological efforts and cause associated costs. Job resources refer to the characteristics that help reduce job demands and associated costs. Job resources are important in assisting the employees in achieving work goals and fulfilling personal development. For disaster volunteers, participating in disaster preparedness requires professional knowledge and skills and is sometimes risky. Professional knowledge and skills reduce the potential risks and help the volunteers fulfill their tasks [26,27,28]. Accidental life insurance serves to reduce potential economic loss. From this perspective, disaster volunteer organizations can provide training, guidance, and accidental life insurance to the disaster volunteers and thereby enhance their willingness to participate in disaster preparedness. The positive association between training and people’s willingness to volunteer in the event of a disaster has been demonstrated by previous studies [29,30]. However, accidental life insurance has not been taken into account. We interviewed some disaster volunteers, and they all emphasized the importance of accidental life insurance, especially for mountain rescue-related activities. 

Based on the analysis above, we propose the following hypotheses:

**Hypothesis** **4.** *Providing accidental life insurance for volunteers has a positive effect on their willingness to participate in disaster preparedness.*

**Hypothesis** **5.** *Providing training for volunteers has a positive effect on their willingness to participate in disaster preparedness.*

**Hypothesis** **6.** *Providing guidance for volunteers has a positive effect on their willingness to participate in disaster preparedness.*

On the other hand, a voluntary organization can “pull” its members to volunteer by providing motivation. Clary and Snyder (1998) proposed Functional Perspective as a way to understand volunteerism [31]. This model assumes that the volunteers’ cognitive evaluation of the individual benefits derived from volunteering influence their decision to volunteer. If the volunteers perceive motivational functions in volunteering, they will show more willingness to volunteer. These functions include altruistic tendencies, protecting the self from its negative features, developing one’s positive aspects, learning practical knowledge and skills, developing career-related skills, and expanding or maintaining social networks. Positive expectations of behavior have also been found to be important correlates of disaster preparedness [32,33]. Spiritual rewards, such as acknowledgement and encouragement from voluntary organization managers or other volunteers, have a positive effect on the self-efficacy of the volunteers [34]. Developing career-related skills or knowledge can help one to win an advantage in job hunting and/or promotion. In Beijing, volunteers can gain certificates of honor by fulfilling their duties. With these certificates, they can enjoy free access to certain libraries, discounts on haircuts, and other forms of social services. College volunteers with a certificate of honor usually receive extra advantages in scholarship competitions. 

Based on the analysis above, we propose the following hypotheses:

**Hypothesis** **7.** *Providing spiritual rewards for volunteers has a positive effect on their willingness to participate in disaster preparedness.*

**Hypothesis** **8.** *Providing certificates of honor for volunteers has a positive effect on their willingness to participate in disaster preparedness.*

### 2.3. Attitudinal Variables

Social Identity Theory (SIT) proposes that the process of organizational identification helps mediate the interaction between self-interest and the group interest [35]. If the volunteers view the achievements of their organizations as beneficial to themselves, they tend to contribute more effort towards engaging in activities [36]. 

Based on the analysis above, we propose the following hypothesis:

**Hypothesis** **9.** *Organizational identification has a positive effect on volunteers’ willingness to participate in disaster preparedness.*

## 3. Materials and Methods

### 3.1. Sampling and Data Collection 

The data used in this analysis were collected through the volunteer network of the Beijing Volunteer Association (BVA), founded in 2008, with annual financing from the Beijing local government. Its major responsibilities include the recruitment, selection, training, and deployment of volunteers. All the disaster volunteers (spontaneous volunteers without professional training not included) and organizations in Beijing are registered on its website. At the time of data collection, there were 140,045 registered disaster volunteers, 71,226 of whom belonged to 537 disaster volunteer organizations. BVA extensively collaborates with all levels of government, universities, research institutes, enterprises, communities, and other NGOs. It has established and co-established a number of sites to provide training, drill performance, information collecting, and reporting for the disaster volunteers. 

Using a convenience sampling method, 1400 volunteers from 537 disaster volunteer organizations were surveyed online using WeChat (a social media platform used by all registered volunteers) (Tencent, Shenzhen, China). Of these, 990 questionnaires were successfully returned for analysis, giving a response rate of 70.7%. The questionnaire included questions on the social background of the volunteers, basic information on the disaster organization to which they belonged, the job demands and resources provided by the organization, the organizational identification of the volunteers, and the volunteers’ willingness to participate in disaster preparedness.

### 3.2. Measurements 

#### 3.2.1. Dependent Variables

Volunteers’ willingness to participate in disaster preparedness was measured by a self-reported five-point scale by asking “How much do you want to participate in disaster preparedness-related activities?” Answers were scored on a five-point Likert scale with the following responses: 1 = “Very Much”, 2 = “A Little Bit”, 3 = “Not Sure”, 4 = “Rather Not”, and 5 = “Absolutely Not”. Questions about the sorts of activities in which volunteers were willing to participate were used as well. The activities mainly included mitigation (e.g., providing information of potential hazards) and capacity building (e.g., providing training for response skills to residents). 

The frequency distribution of the dependent variable is shown in Table 1. Overall, 28.08% of the respondents indicated that they were very much ready to participate in voluntary disaster preparedness; 14.65% of the respondents showed a little bit of interest; 31.41% of the respondents answered “Not Sure”; and the rest answered “Rather not” or “Absolutely not”. 

**Figure 1:**
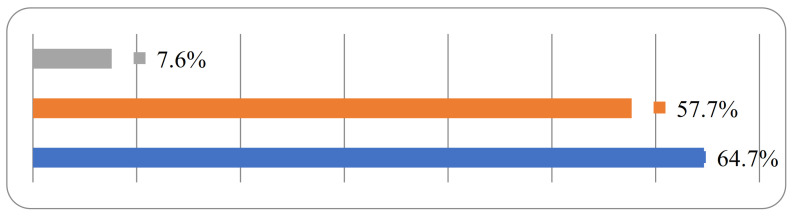
Distribution of willingness to participate in voluntary disaster preparedness activities. Note: Gray bar stands for emergency information report. Orange bar stands for drill organizing activities. Blue bar stands for disaster knowledge and skill training activities.

Figure 1 presents the three major types of disaster preparedness activities the respondents were willing to participate in. It can be seen that the most popular activity was disaster knowledge and skill training (64.7%). Drill organizing ranked second (57.7%). The results reflect a strong need for practical disaster response knowledge and skills. 

#### 3.2.2. Independent Variable

There were three sets of independent variables in this analysis. The first set consisted of social background variables, including gender, education, and political status (whether a member of CCP). The detailed distribution of these variables is shown in Table 2.

The second set consisted of organizational variables, which consisted of how often the volunteer organization provided the members with training and guidance to ensure adequate capacity, whether the volunteer organization purchased accidental life insurance for the members to reduce potential risks, and how often voluntary organizations offered spiritual rewards or official certificates of honor for the volunteers. A detailed distribution of the organizational variables is shown in Table 3.

The third set consisted of attitudinal variables. The organizational identification of the disaster volunteers was measured by a self-reported scale by asking “Do you think becoming a disaster volunteer is your own choice?”, ”Do you think being a disaster volunteer is important to you?”, ”Do you care about being a disaster volunteer?”, and ”Do you think giving up the identity of a disaster volunteer would have a negative impact on you?”. These items were scored on a five-point Likert scale: 1 = “Not at all”, 2= “Little”, 3 = “Hard to say”, 4 = “Some”, and 5 = “Very much”. The mean of the identity degrees was used as the volunteers’ willingness predictor (Cronbach’s alpha = 0.631). The organizational identification had a mean value of 2.08 with a standard deviation of 0.63, a minimum value of 1, and a maximum value of 4.75 (Table 4). 

### 3.3. Data Analysis Methods

Linear regression models were constructed using willingness to voluntarily participate in disaster preparedness as the dependent variable. The statistical software Stata 15.0 (StataCorp LLC, College Station, TX, USA) was used for data analysis.

## 4. Results

Table 5 presents the results from the regression analyses. Model 1 only included the social background variables to quantify their influence on willingness to participate. Compared to male volunteers, female volunteers had a stronger willingness to participate in disaster preparedness (β = 0.52, *p* < 0.001), and thus Hypothesis 1 is rejected. Being a Communist Party member had a positive effect on volunteers’ willingness to participate in disaster preparedness (β = −0.22, *p* < 0.05), and thus Hypothesis 3 is supported. No significant relation was shown between education and willingness to participate and thus Hypothesis 2 is rejected. This may be partly because most of the respondents had received a college degree or above (89.9%).

Model 2 added organizational variables as independent variables. The results show that providing accidental life insurance for the volunteers had a positive effect on willingness to participate (β = 0.34, *p* < 0.001). This outcome supports Hypothesis 6. Specifically, when other conditions remained unchanged, with accidental life insurance provided by the organization, disaster volunteers’ willingness to participate increased by 0.34 units on average. No significant relation was found between training, guidance, certificates, or spiritual rewards and willingness to participate, and thus Hypotheses 5, 6, 7, and 8 are rejected. The effect of gender and political status was still significant, with a minor change in gender. 

Model 3 added attitudinal factors as independent variables. The results show that organizational identification was significantly positively related to participation willingness (β = 0.31, *p* < 0.001), and thus Hypothesis 9 is supported. Specifically, when other conditions remained unchanged, for every one-unit increase in organizational identification of disaster volunteers, their willingness to participate increased by 0.31 units on average. The effects of gender and political status were still significant. The effects of guidance, certificates, or spiritual rewards on willingness to participate were still insignificant. There are two possible explanations for this. One is that the forms and/or content of these measures may not have been sufficiently well designed. Another possible explanation is that guidance, certificates, or spiritual rewards failed to satisfy the actual needs of the volunteers. It is noteworthy that training was partly related to willingness to participate. The outcomes revealed a result opposite to our Hypothesis 5. Provision of training was negatively associated with willingness to participate in disaster preparedness, indicating a low quality of training. More training caused a higher turnover of volunteers.

## 5. Conclusions

This study aimed to discover the key factors affecting disaster volunteers’ willingness to participate in disaster preparedness. Based on prior literature, it categorized these factors into social background, organizational, and attitudinal factors, and it proposed the “pulling” and “pushing” factors of organizations for the first time.

Based on a quantitative survey from 990 registered disaster volunteers across Beijing, we found a weak willingness to participate. Only 28.08% of the respondents indicated that they were “very much” ready to participate in voluntary disaster preparedness, and 14.65% showed “a little bit” of interest. These findings are in agreement with prior studies [3,4,5,37]. 

Linear regression models were conducted and the following conclusions were found: (1) Disaster volunteers’ social background variables were associated with their willingness to participate in disaster preparedness. Contrary to our hypothesis, female volunteers were more willing to participate in disaster preparedness than male volunteers. It could be inferred that femininity did not limit the females’ efforts in disaster preparedness. On the contrary, females may be more caring about the well-being of other people and more devoted to their communities. Being a CCP member was positively associated with the volunteers’ willingness to participate in disaster preparedness. As mentioned before, people with traditional virtues, such as being altruistic and devoted to community development, usually have an advantage when applying to become a CCP member. Education had no significant effect on the volunteers’ willingness to participate. This may be because most of the respondents of this survey had received a college degree or above (89.9%). (2) Most of the organizational variables were not significantly associated with the volunteers’ willingness to participate in disaster preparedness. Among these factors, providing accidental life insurance for the volunteers had a positive effect on their willingness to participate. Provision of accidental life insurance would reduce potential loss for volunteers, as some disaster preparedness activities are risky. Contrary to our hypothesis, provision of training had an opposite effect. This might indicate that a low quality of training caused the turnover of the volunteers. Guidance, certificates, and spiritual rewards had no significant relation to willingness to participate. This indicates the poor quality of the motivating measures or their failure to satisfy the actual needs of the volunteers. (3) Organizational identification was positively associated with the volunteers’ willingness to participate and thus supported our hypothesis.

According to the conclusions above, we propose the following suggestions. First, organizations should enroll more CCP members as volunteers, considering their stronger willingness to participate and their commitment to community safety in general. Second, more family support measures should be taken to support female volunteers. Third, all disaster organizations are advised to provide accidental life insurance for volunteers who participate in disaster preparedness. This insurance will provide compensation for potential loss during disaster preparedness activities and thus reduce the safety concerns of the volunteers. Fourth, organizations should pay more attention to culture building. Culture building will enhance organizational identification and thus improve the volunteers’ willingness to participate. Last but not least, the quality and content of the training, guidance, certificates, and spiritual rewards provided by the volunteer organizations should be improved. The real motivations behind volunteering should be investigated so that more motivating plans can be made accordingly.

Although this study provides a useful exploration of the correlations between social background, organizational, and attitudinal variables and the volunteers’ willingness to participate in disaster preparedness, it has some deficiencies. The findings underscore the importance of social support, such as family support and work unit support. Moreover, the situational variables were not discussed here, which might introduce bias into the results. These supporting factors and situational variables should be taken into account for further investigation. Last but not least, this study adopted a cross-sectional survey data methodology to examine the hypotheses, which limited the assessment of causal inferences [38]. Future studies should use multi-source, multi-level, or longitudinal data.

## Figures and Tables

**Table 1:** Frequency distribution of the dependent variables (%).

Answers	Willingness to Participate
Very Much	28.08
A Little Bit	14.65
Not Sure	31.41
Rather Not	16.87
Absolutely Not	8.99
Total	100

**Table 2:** Social background variables of the respondents (%).

Variables	Freq.	Percent	Cum.
**Gender**			
Female	641	64.75	64.75
Male	349	35.25	100.00
**Education**			
Middle school and below	17	1.72	1.72
High school	83	8.38	10.10
College and above	890	89.90	100.00
**Party**			
CCP	412	41.62	41.62
Others	578	58.38	100.00

**Table 3:** Organizational variables of the respondents (%).

Variables	Freq.	Percent	Cum.
**Training Provided**			
Frequently	341	34.44	34.44
Medium	449	45.35	79.80
Seldom	200	20.20	100.00
**Guidance Provided**			
Frequently	288	29.09	29.09
Medium	441	44.55	73.64
Seldom	261	26.36	100.0
**Accidental Life Insurance Provided**			
Yes	443	44.75	44.75
No	547	55.25	100.00
**Certification Provided**			
Frequently	307	31.01	31.01
Medium	424	42.83	73.84
Seldom	259	26.16	100.0
**Spiritual Reward Provided**			
Frequently	195	19.70	19.70
Medium	360	36.36	56.06
Seldom	435	43.94	100.0

**Table 4:** Attitudinal variables of the respondents (%).

Variable	Observation	Mean	SD	Min	Max
Organizational identification	990	2.42	0.50	1	4.75

**Table 5:** Regression results.

Variables	Model 1	Model 2	Model 3
Gender (Female = 1)	0.52 ***	0.45 ***	0.43 ***
Party (not CCP member = 1)	−0.22 **	−0.22 **	−0.23 **
Education (Middle school and below as reference			
High school	−0.19	−0.21	−0.14
College and above	−0.19	−0.25	−0.19
Accidental Life Provided (Yes = 1)		0.34 ***	0.33 ***
Training Provided (Frequently as reference)			
Medium		−0.02	−0.02
Seldom		0.51	0.46 *
Guidance Provided (Frequently as reference)			
Medium		0.29	0.24
Seldom		0.03	−0.03
Certificates Provided (Frequently as reference)			
Medium		−0.04	−0.07
Seldom		−0.26	−0.28
Spiritual Rewards Provided (Frequently as reference)			
Medium		0.04	−0.01
Seldom		0.05	−0.02
Organizational Identification			0.31 ***
N	990	990	990
R^2^	0.15	0.16	0.17

Notes: Standard errors in parentheses. ***, **, and * indicate statistical significance at the 1%, 5%, and 10% levels, respectively.

## Data Availability

We signed a non-disclosure agreement with the BVA regarding the data from this survey.
